# *CmWRKY15* Facilitates *Alternaria tenuissima* Infection of Chrysanthemum

**DOI:** 10.1371/journal.pone.0143349

**Published:** 2015-11-24

**Authors:** Qingqing Fan, Aiping Song, Jingjing Xin, Sumei Chen, Jiafu Jiang, Yinjie Wang, Xiran Li, Fadi Chen

**Affiliations:** 1 College of Horticulture, Nanjing Agricultural University, Nanjing, China; 2 Jiangsu Province Engineering Lab for Modern Facility Agriculture Technology & Equipment, Nanjing, China; Oak Ridge National Laboratory, UNITED STATES

## Abstract

Abscisic acid (ABA) has an important role in the responses of plants to pathogens due to its ability to induce stomatal closure and interact with salicylic acid (SA) and jasmonic acid (JA). WRKY transcription factors serve as antagonistic or synergistic regulators in the response of plants to a variety of pathogens. Here, we demonstrated that CmWRKY15, a group IIa WRKY family member, was not transcriptionally activated in yeast cells. Subcellular localization experiments in which onion epidermal cells were transiently transfected with CmWRKY15 indicated that CmWRKY15 localized to the nucleus *in vivo*. The expression of *CmWRKY15* could be markedly induced by the presence of *Alternaria tenuissima* inoculum in chrysanthemum. Furthermore, the disease severity index (DSI) data of *CmWRKY15*-overexpressing plants indicated that *CmWRKY15* overexpression enhanced the susceptibility of chrysanthemum to *A*. *tenuissima* infection compared to controls. To illustrate the mechanisms by which *CmWRKY15* regulates the response to *A*. *tenuissima* inoculation, the expression levels of ABA-responsive and ABA signaling genes, such as *ABF4*, *ABI4*, *ABI5*, *MYB2*, *RAB18*, *DREB1A*, *DREB2A*, *PYL2*, *PP2C*, *RCAR1*, *SnRK2*.*2*, *SnRK2*.*3*, *NCED3A*, *NCED3B*, *GTG1*, *AKT1*, *AKT2*, *KAT1*, *KAT2*, and *KC1*were compared between transgenic plants and controls. In summary, our data suggest that *CmWRKY15* might facilitate *A*. *tenuissima* infection by antagonistically regulating the expression of ABA-responsive genes and genes involved in ABA signaling, either directly or indirectly.

## Introduction

Recently, the complex mechanism by which abscisic acid (ABA) responds to pathogens has been extensively studied and reviewed [1,2]. Stomatal closure is commonly regarded as a defense mechanism that can prevent bacterial pathogen infection [3]. As a result, ABA can have a positive effect on disease resistance through its regulation of stomatal movements. Furthermore, ABA has emerged as an important regulator of interactions with other hormones involved in plant defense mechanisms [1].

Plants must defend themselves against diverse types of pathogens and must be capable of enduring pathogen-induced stress conditions. A number of plant hormones, such as salicylic acid (SA), jasmonic acid (JA), and ethylene (ET), are associated with pathogen defense mechanisms [4]. It is generally believed that SA behaves as a critical regulator of defense responses against biotrophic pathogens such as *Oidium neolycopersici* and *Hyaloperonospora parasitica* [5]. JA- and ET-associated defense mechanisms yield resistance against necrotrophic pathogens, such as *Botrytis cinerea*, *Alternaria brassicicola and Plectosphaerella cucumerina* [6,7]. Several studies have suggested that ABA can interact antagonistically or synergistically with pathogen infection-related signaling pathways involving SA, JA and ET. Although ABA negatively regulates SA, it can enhance resistance to necrotrophic pathogen attacks by increasing JA biosynthesis [6]. Thus, these results demonstrate that ABA can function in plant immunity by promoting one defense pathway while impairing another signaling pathway, allowing plants to integrate and fine-tune their defense mechanisms against diverse types of pathogens and colonization attempts [8].

Moreover, an ever-growing body of evidence has revealed that ABA can act either synergistically or antagonistically with host defense mechanisms against pathogens. Mounting evidence supports the notion that ABA represses resistance to pathogens. For example, ABA accumulation was found to increase the susceptibility of barley to the hemi-biotroph *Magnaporthe oryzae* [9]. In addition, three *Arabidopsis* ABA mutants, *aba2-12*, *aao3-2*, and *abi4*, were found to exhibit enhanced stress-adaptive responses to the necrotroph *B*. *cinerea*; ABA biosynthesis was impaired in both *aba2-12* and *aao3-2*, whereas *abi4* was insensitive to ABA. These three ABA mutants exhibited increased susceptibility to the necrotroph *Alternaria brassicicola* [7]. Thus, ABA fine-tuned and enhanced the immune response to pathogen attacks. Similarly, exogenous ABA application was found to provide rice (*Oryza sativa*) with the capability to resist brown spot disease caused by *Cochliobolus miyabeanus* [2]. Interestingly, exogenously administered ABA also enhanced the basal defense of tomato (*Solanum lycopersicon L*.) against *Alternaria solani* [10]. Taken together, these data indicate that the role of ABA in regulating pathogen-associated pathways is multifaceted. Moreover, the mechanism of interaction between WRKY transcription factors and ABA in response to pathogens remains an open question.

The WRKY family was named based on the WRKY domain, consisting of a conserved WRKYGQK heptapeptide at the N-terminus along with a C_2_H_2_- or C_2_HC-type zinc finger motif [11]. Additional research has shown that WRKY transcription factors can bind to the W box (TTGACY) sequence, thus allowing interaction with the promoters of target genes. The WRKY transcription factor family has been identified in many species, such as *A*. *thaliana*, *Glycine max*, *Brachypodium distachyon* and *Chrysanthemum morifolium* [11,12]. In *Arabidopsis*, the WRKY transcription factor family consists of over 74 members, and 15 WRKY genes were isolated from chrysanthemum [13]. Five group II *WRKY* genes have been recently cloned from chrysanthemum, one of which inhibits aphid population growth [14]. In this study, we focused on *CmWRKY15*, which is homologous to *AtWRKY40* in *Arabidopsis* [13].

There are three functionally and structurally homologous WRKY transcription factors in *A*. *thaliana*: WRKY18, WRKY40 and WRKY60. These transcription factors trigger a complex pattern of activity that is implicated in pathogen infection and ABA-associated signaling [15]. WRKY40 acts as a negative or positive regulator of plant defense mechanisms against pathogens. For example, WRKY18 and WRKY40, which are closely related, worsened biotrophic powdery mildew infection of *Arabidopsis* due to their negative effects on pre-infection mechanisms of host defense [15]. In contrast, WRKY18 and WRKY40 were found to act synergistically in effector-triggered immunity, as the *wrky18wrky40* double mutant showed increased susceptibility to the bacterial pathogen *Pseudomonas syringae* DC3000 releasing the effector *AvrRPS4* [16].

Over the past few decades, it has become increasingly clear that WRKY40 predominates at the nodes of ABA-responsive signaling networks, where it serves as an antagonistic regulator that directly suppresses a group of ABA-responsive genes [17, 18]. Genes involved in the ABA response and in ABA signaling include *ABF4*, *ABI1*, *ABI2*, *ABI4*, *ABI5*, *DREB1A*, *DREB2A*, *MYB2*, *PYL2⁄ RCAR13*, *PYL2⁄ RCAR11*, *RAB18*, *PYL2⁄ RCAR9*, *PYL2⁄RCAR7*, *SnRK2*.*2* and *SnRK2*.*3*, the expression of which is distinctly altered in *WRKY40* knockout mutants [18]. Indeed, WRKY40 was found to directly inhibit *ABI5* expression and also downregulate *ABF4*, *ABI4*, *ABI5*, and *MYB2* [17,18]. However, the underlying mechanisms of WRKY40-mediated regulation of the ABA signaling pathway remain unclear.

Chrysanthemum (*Chrysanthemum morifolium*), which is one of the most famous cut flowers globally, has high ornamental value and occupies an irreplaceable position in international flower commerce [19]. Black spot disease, one of the most harmful diseases of chrysanthemum, is caused by the necrotrophic fungus *Alternaria*. High humidity and warm conditions result in serious disease, which causes year-round reductions in greenhouse-based yields. Spraying broad-spectrum fungicide not only leads to increases in cost and energy consumption but also causes severe environmental contamination and alters pesticide resistance. Hence, to alleviate pathogen damage, more research is needed on the molecular mechanisms of *Alternaria tenuissima* infection of chrysanthemum. Previous research suggests that *WRKY* genes act as central regulatory factors in plant disease immunity [20,21]. In this study, we investigated the mechanisms by which *CmWRKY15* regulates the ABA response to *A*. *tenuissima* in chrysanthemum.

## Material and Methods

### Plant materials and growth conditions

We used the chrysanthemum cultivar ‘Jinba’, which was obtained from the Chrysanthemum Germplasm Resource Conservation Center, Nanjing Agricultural University, China. Uniform cuttings were propagated in pots using a 1:1 (v/v) mixture of soil and vermiculite and cultivated in a greenhouse (day/night temperature of 25/18°C, a light/dark photoperiod of 14/10 h, a light intensity of 50 μmol m^-2^s^-1^ and a relative humidity of 70%).

### Phylogenetic analysis of homologous CmWRKY15 sequences

The CmWRKY15 amino acid sequence was aligned with its homologues using DNAman 5.2.2 software and BLAST (http://www.ncbi.nlm.gov/blast). The conserved WRKY domains of *CmWRKY15* homologues were acquired from the NCBI database (http://www.ncbi.nlm.nih.gov). We performed various sequence alignments of the WRKY domains from different species using ClustalW [22]. To obtain better classifications of the multiple branches, we generated phylogenetic trees containing eighteen representative orthologs from the CmWRKY15 alignment using the MEGA5 program [23] following the neighbor-joining method with 1000 bootstrap replicates.

### Transcriptional activity analysis of CmWRKY15

The transcriptional activation of *CmWRKY15* was evaluated using a yeast assay system [24]. The ORF of *CmWRKY15* without the stop codon was amplified using the Phusion^®^ High Fidelity PCR Kit (New England Biolabs, Ipswich, MA, USA) with the primer pair CmWRKY15-GATE-SAL-F/CmWRKY15-GATE-NOT-R (S1 Table). The PCR products were cloned into the pENTR^™^1A vector (Invitrogen, Carlsbad, CA, USA) by *Sal* I/*Not* I double digestion and ligation. Sequencing was carried out to confirm the presence of *CmWRKY15* in the construct. pENTR^™^1A-*CmWRKY15* and pDEST-GBKT7 were recombined to form pDEST-GBKT7-*CmWRKY15* using LR Clonase^™^ II enzyme mix (Invitrogen). pCL1 and pDEST-GBKT7 plasmids were used to create positive and negative control strains, respectively. pDEST-GBKT7-*CmWRKY15*, pCL1 and pGBKT7 were introduced into *Saccharomyces cerevisiae* strain Y2HGold (Clontech) according to the manufacturer’s instructions. Selection of transformants carrying either pGBKT7-*CmWRKY15* or pGBKT7 was performed using SD/-Trp medium, whereas pCL1 was selected using SD/-Leu medium. Y2H cells containing pCL1 (positive control) can persist on SD/-His-Ade medium. Conversely, Y2H cells containing pGBKT7 (negative control) cannot grow on this medium. We utilized a luminescence assay to assess the transcriptional activity of CmWRKY15. The CmWRKY15 ORF was amplified by PCR using the primer set CmWRKY15-GATE-F/R (S1 Table) containing *Bam HI* and *Not I* sites to obtain the 35S::GAL4DB-CmWRKY15 fusion construct. Further, the amplified DNA fragment was inserted into the pENTRTM1A dual selection vector (Invitrogen) to obtain pENTRTM1A-CmWRKY15, which was confirmed by sequencing. We recombined this plasmid with 35S::GAL4DB to generate 35S::GAL4DB-CmWRKY15 via the LR reaction (Invitrogen). *Arabidopsis* protoplasts were obtained and transfected according to the protocol previously described by Yoo et al [25]. We transfected 7.5 μg of 35S::GAL4DB-AtARF5, 35S::GAL4DB or 35S::GAL4DB-CmWRKY15 with 7.5 μg of 5X GAL4-LUC, which contains the luciferase reporter gene driven by five copies of the GAL4 binding element. The luciferase activity was assessed as described previously [26]. Three independent experiments were performed.

### Subcellular localization of CmWRKY15

To assess the subcellular localization of CmWRKY15, we performed transient transfection of onion epidermal cells [27]. To generate the *p35S*::*GFP*-*CmWRKY* plasmid, we first generated a green fluorescent protein (GFP)-*CmWRKY15* fusion construct using the plasmid pENTR^™^1A-*CmWRKY15*, which is based on pMDC43, and LR Clonase^™^ II enzyme mix (Invitrogen). The *p35S*::*GFP*-*CmWRKY* construct and the empty pMDC43 vector, which was used as a marker for transgene expression, were transiently transfected into onion epidermal cells. Confocal laser microscopy was used to monitor GFP expression.

### Transformation of chrysanthemum

We acquired *CmWRKY15-*overexpressing chrysanthemum transformants to further analyze the function of *CmWRKY15*. The *Agrobacterium tumefaciens* strain EHA105 was transformed with the plasmid 35S::*CmWRKY15* using the freeze-thaw method. Transformation of chrysanthemum was performed as described above [14]. Leaf discs (5 mm in diameter) obtained from mature plants of ‘Jinba’ cultured *in vitro* were used as explants. Initially, we selected transformants by cultivating them on a medium containing 8 mg L^-1^ hygromycin. After regeneration, RNA was extracted from the putative transgenic chrysanthemum and wild type (WT) plants using the RNAiso reagent (TaKaRa). Extracted RNA was digested with RNase-free DNase I (TaKaRa) and reverse transcribed using M-MLV reverse transcriptase (TaKaRa). The transcript level of *CmWRKY15* was assessed with quantitative real-time PCR (qPCR) analysis using SYBR^®^ Green (TaKaRa) and the primer pair *CmWRKY15*-DL-F/R (S1 Table). The primer pair CmEF1α-F/R was used to amplify the reference gene *CmEF1α*. All qPCRs were run on a Mastercycler ep realplex device (Eppendorf, Hamburg, Germany). Fold changes in expression were calculated using the 2^−ΔΔCt^ method [28].

### *A*. *tenuissima* infection of transgenic chrysanthemum and wildtype plants

Groups of 50 cuttings each were taken from transgenic chrysanthemum (W15) and non-transgenic chrysanthemum (‘Jinba’, WT) plants for *A*. *tenuissima* resistance detection. Groups of 10 cuttings each were used as controls with watering. Cuttings were maintained at 22 ± 3°C under a 14/10 h light/dark photoperiod in aerated water for 20 d. *A*. *tenuissima* conidia were isolated from naturally infected diseased chrysanthemum plants and cultured on potato dextrose agar medium at 25°C in the dark [29]. Host resistance was assessed using the method described by Deng et al [30]. After a 15-d culture, the conidia were suspended in sterile distilled water, and an aqueous suspension of 10^6^ spores per ml was prepared with a few drops of Triton X-100 added as a wetting agent. This mixture was sprayed over the seedlings produced from 20-day-old root cuttings. Each treatment comprised 50 seedlings of each of transgenic chrysanthemum plant and wild type plants. Mock treatments were carried out with 10-μl droplets of sterile distilled water. After inoculation, the seedlings produced from 20-day-old root cuttings were maintained at 100% relative humidity in the dark at a temperature of 25°C for 48 h and then moved to a chamber held at a constant temperature of 25°C with a 16/8 h photoperiod, a light intensity of 50 μmol m^−2^ s^−1^ and a relative humidity of 90%. Three seedlings each of W15-1, W15-2 and WT were used for leaf sampling under each treatment at 0 h, 6 h, 24 h, 48 h, 72 h, 96 h and 120 h after exposure to *A*. *tenuissima*. We removed the third true leaf from each of the three seedlings of the same line. Each experiment included three biological replicates. Samples collected at defined time points for each treatment were pooled for RNA extraction. The proportion of plants showing disease symptoms (PPD) was measured 14 d after inoculation. The mean lesion size (MLS) at this time point was used to determine the resistance level according to Xu [29]. Fourteen days after inoculation, plant disease severity was assessed on a 0–5 scale based on the percentage of leaf area that was symptomatic, such that 0 represented 0%, 1 represented up to 10%, 2 represented 11–25%, 3 represented 26–50%, 4 represented 51–75%, and 5 represented more than 75% or an abscised leaf. Chlorotic portions of leaves were considered infected. The disease severity index (DSI) for each plant was calculated using the following formula: (no. leaves in class × severity class)/(no. leaves examined × maximum severity class) × 100. The host plant response was classified based on the mean DSI such that 0 was immune (I), 1–10 was highly resistant (HR), 11–20 was resistant (R), 21–30 was moderately resistant (MR), 31–45 was moderately susceptible (MS), 46–70 was susceptible (S), and over 70 was highly susceptible (HS).

### Measurement of ABA content

Uniform cuttings were propagated in pots using a 1:1 (v/v) mixture of soil and vermiculite and cultivated in a greenhouse (day/night temperature of 25/18°C, a light/dark photoperiod of 14/10 h, a light intensity of 50 μmol m^-2^s^-1^ and a relative humidity of 70%). Each treatment comprised 10 seedlings of each of transgenic chrysanthemum line and wild type plants. Mock treatments were carried out with 10-μl droplets of sterile distilled water. After inoculation, seedlings produced from 20-day-old root cuttings were maintained at 100% relative humidity in the dark at a temperature of 25°C for 48 h and then moved to a chamber held at a constant temperature of 25°C with a 16/8 h photoperiod, a light intensity of 50 μmol m^−2^ s^−1^ and a relative humidity of 90%. The leaves of three seedlings from each treatment were sampled at 0 h and 24 h after exposure to *A*. *tenuissima*. We removed the third true leaf from each of the three plants of the same line. Each experiment included three biological replicates. Leaves from two transgenic lines and wildtype ‘Jinba’ were frozen in liquid nitrogen and then stored at -80°C until they were used in experiments. The ABA content was analyzed by ultra-performance liquid chromatography (UPLC). The extraction was performed based on a previously published protocol [31]. In brief, 1 g of lyophilized plant material was immersed in 10 ml of cold 80% (v/v) methanol with constant shaking at 4°C for 12 h. After mechanical homogenization, the extract was clarified by centrifugation (10,000 g for 15 min) followed by incubation at 4°C with constant shaking for 1 h with the addition of 0.2 g of PVPP; the extract was then centrifuged as described previously. The supernatant was collected and passed through a C^18^ Sep-Pak cartridge (Waters Corp., Milford, MA, USA). An aliquot of the eluate was dried under a stream of nitrogen gas, and the residue was dissolved in 1 ml of methanol. ABA was quantified using an Agilent 1290 Infinity UPLC system.

### Response of *CmWRKY15* expression to changes in *A*. *tenuissima* stress-related genes in *CmWRKY15*-transformed chrysanthemum

To identify the regulatory mechanisms of *CmWRKY15* in response to *A*. *tenuissima* infection, cDNA was synthesized using RNA from the leaves of WT and transgenic chrysanthemum plants. ABA-responsive genes were monitored, including *ABF4*, *ABI4*, *ABI5*, *RAB18*, *DREB1A*, *DREB2A*, *PYL2*, *PP2C*, *SnRK2*.*2*, *SnRK2*.*3*, *RCAR1*, *KAT1*, *KAT2*, *AKT1*, *AKT2*, and *KC1*. Moreover, genes involved in ABA signaling were also monitored, including *MYB2*, *NCED3A*, *NCED3B* and *GTG1*. The sequences of all relevant primers are listed in S1 Table.

### Statistical analysis

One-way analysis of variance was used to identify significant differences among treatments. Multiple comparisons were performed using Tukey’s multiple range test (p = 0.05). All statistical analyses were carried out using SPSS v17.0 (SPSS Inc., Chicago, IL, USA).

## Results

### Phylogenetic analysis of homologous CmWRKY15 sequences

**Fig 1:**
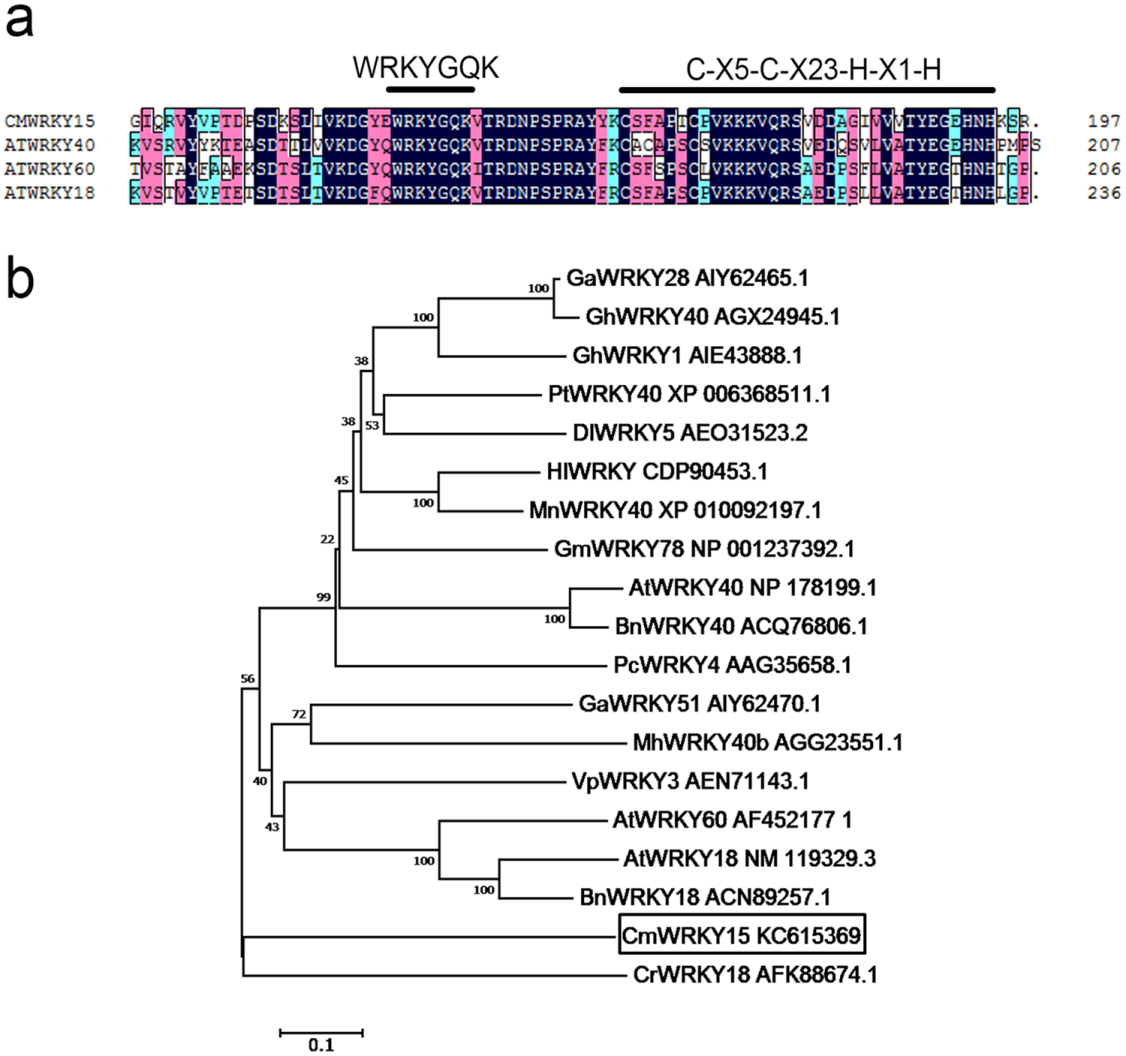
Deduced peptide sequences of CmWRKY15 and other WRKY proteins. **a** Alignment of the putative amino acid sequence of CmWRKY15 with the amino acid sequences of homologous proteins. Features of the sequence include a WRKY domain (WRKYGQK) and a C_2_H_2_ zinc finger domain (both highlighted by lines above the alignment). **b** A phylogenetic tree shows homologues of CmWRKY15 and WRKY proteins from other species. ClustalW was used to align the amino acid sequences, and the neighbor-joining method was used to build the phylogenetic tree with MEGA 5.0. The accession numbers for the sequences are listed below. AtWRKY40 (NP_178199.1), AtWRKY60 (AAL50787.1), AtWRKY18 (Q9C5T4.2), GarWRKY28 (AIY62465.1), GarWRKY51 (AIY62470.1), PtWRKY40 (XP_006368511.1), BnWRKY40 (ACQ76806.1), CrWRKY18 (AFK88674.1), VpWRKY3 (AEN71143.1), PcWRKY4 (AAG35658.1), MhWRKY40b (AGG23551.1), GmWRKY78 (NP_001237392.1), HvWRKY7 (ABI13373.1), GhWRKY40 (AGX24945.1), HlWRKY (CDP90453.1), MnWRKY40 (XP_010092197.1), DlWRKY5 (AEO31523.2). CmWRKY15 is *boxed*.

CmWRKY15, AtWRKY40, AtWRKY18, and AtWRKY60 contained one WRKY domain (WRKYGQK) and one C_2_H_2_ zinc finger motif (C-X5-C-X23-H-X1-H) (Fig 1a). Phylogenetic analysis showed that CmWRKY15 was most closely related to CrWRKY18 and showed high similarity to AtWRKY40, AtWRKY18 and AtWRKY60 in *Arabidopsis*. Moreover, CmWRKY15 also showed high similarity to GhWRKY40, VpWRKY3, BnWRKY18, BnWRKY40, PcWRKY4, GarWRKY28 and GarWRKY51 (Fig 1b).

### Subcellular localization of CmWRKY15

**Fig 2:**
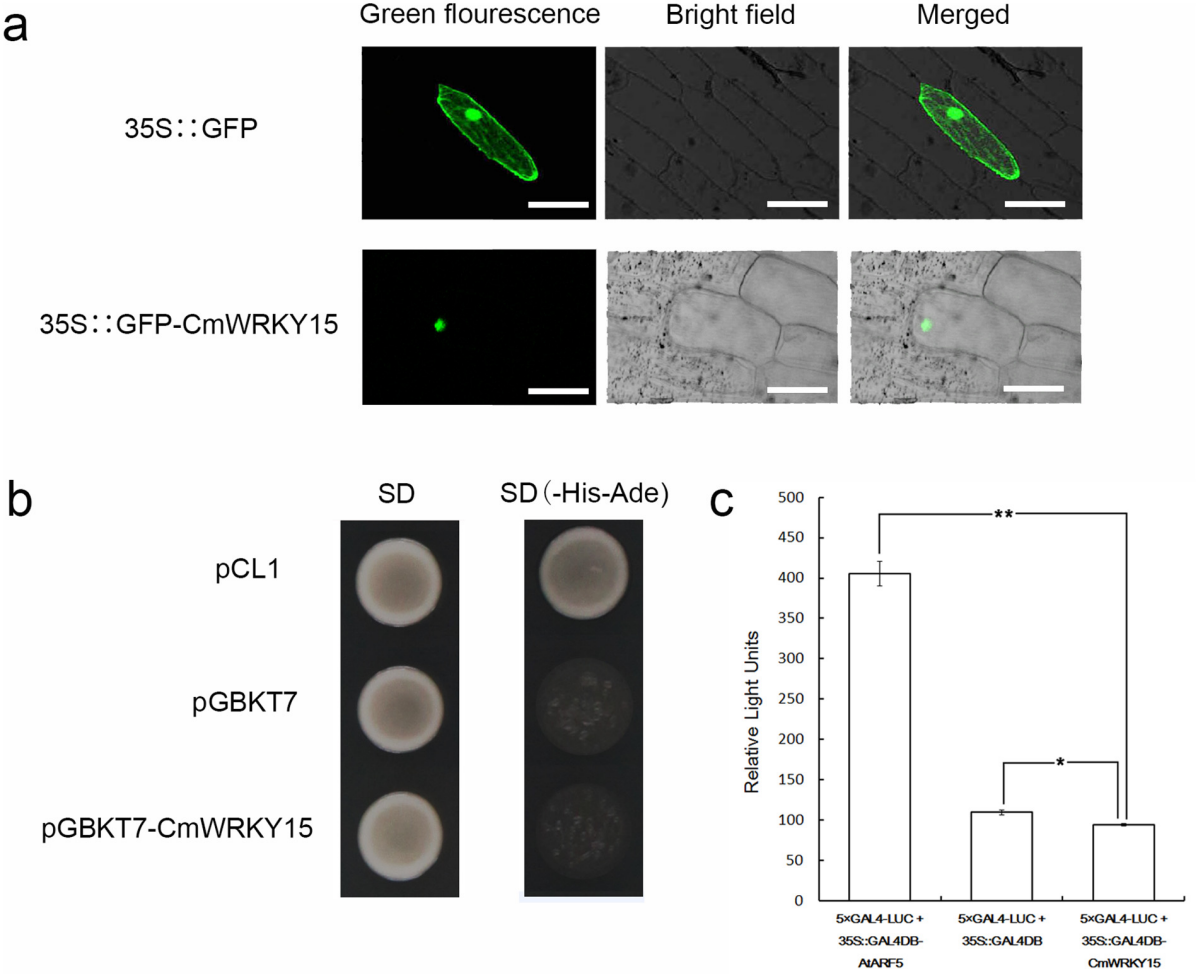
Subcellular localization and transactivation analysis of CmWRKY15. **a** Subcellular localization of CmWRKY15. **b** The analysis of CmWRKY15 transcriptional activity was carried out using a yeast assay system. The positive control cells (Y2H cells containing pCL1) were able to grow on SD/-His-Ade medium. Conversely, the negative control cells (Y2H cells containing pGBKT7) could not grow on this medium. **c** Relative luciferase activities in *Arabidopsis* mesophyll protoplasts after transfection with 35S::GAL4DB-CmWRKY15.

The 35S::GFP-*CmWRKY15* construct and a positive vector harboring only 35S::GFP were introduced into onion epidermal cells via particle bombardment. Onion epidermal cells expressing 35S::GFP showed GFP fluorescence throughout the cells (Fig 2a). In contrast, GFP fluorescence was localized solely in the nuclei of the onion epidermal cells transformed with the 35S::GFP-*CmWRKY15* fusion protein (Fig 2a). These results indicated that *CmWRKY15* localized to the nucleus *in vivo*.

### Transrepression assay of CmWRKY15

Transcriptional activation of CmWRKY15 was evaluated using a yeast one-hybrid assay. The negative control pGBKT7 and the pGBKT7-*CmWRKY15* construct were both incapable of growing on SD/-His-Ade medium, whereas the pCL1 positive control grew normally (Fig 2b). These results suggest that CmWRKY15 was not transcriptionally active in yeast cells.

To further understand the transactivation function of CmWRKY15, *Arabidopsis* protoplasts were transfected with the plasmid encoding CmWRKY15 together with a reporter plasmid. The results (Fig 2c) indicate that 35S::GAL4DB-AtARF5 showed a significant increase in relative LUC units (RLUs) compared to 35S::GAL4DB-CmWRKY15 in *Arabidopsis* protoplasts (*P*<0.01), whereas 35S::GAL4DB-CmWRKY15 showed a decrease in RLUs compared with 35S::GAL4DB (*P*<0.05) (Fig 2c). These results indicate that CmWRKY15 functions as a transcriptional repressor.

### *CmWRKY15* overexpression enhanced the susceptibility of chrysanthemum to *A*. *tenuissima*

**Fig 3:**
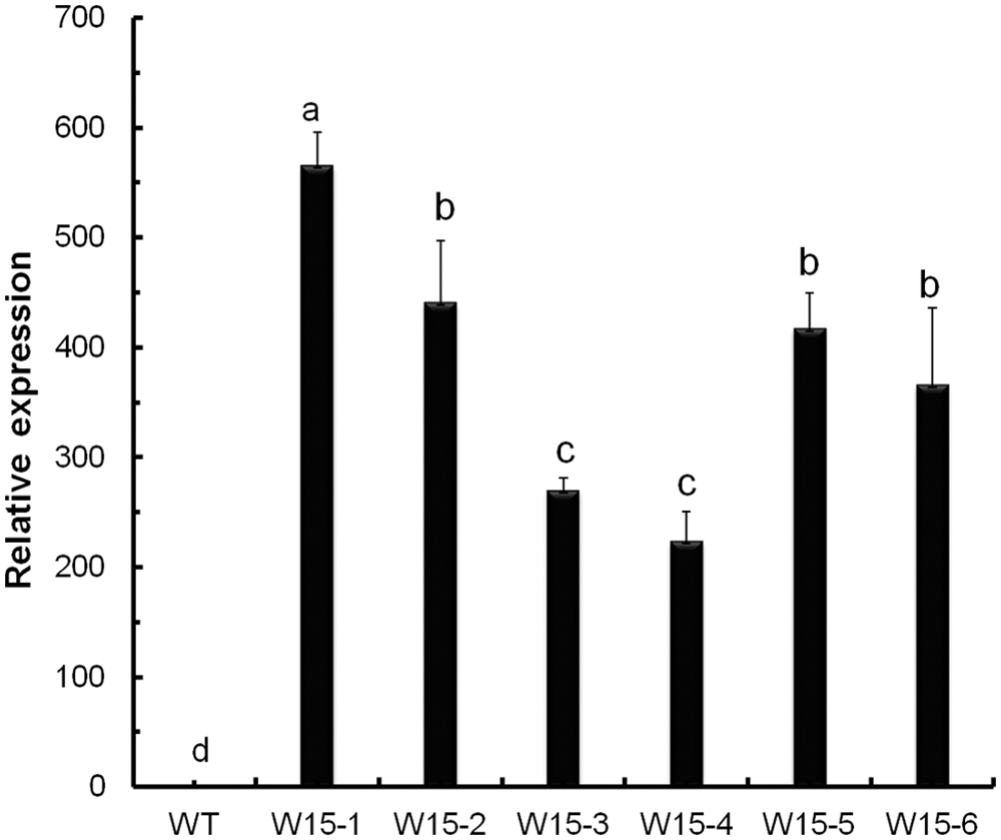
The relative expression level of the *CmWRKY15* gene in transgenic plants. Expression levels of *CmWRKY15* in wildtype ‘Jinba’ and transgenic chrysanthemum lines overexpressing *CmWRKY15*.

We used transgenic chrysanthemum lines overexpressing *CmWRKY15*, the relative expression levels of which were determined using quantitative real time PCR (qPCR). In the overexpressing plants (W15-1, W15-2, W15-5 and W15-6), the expression levels of *CmWRKY15* were markedly higher than those in the nontransformed controls (Fig 3).

**Fig 4:**
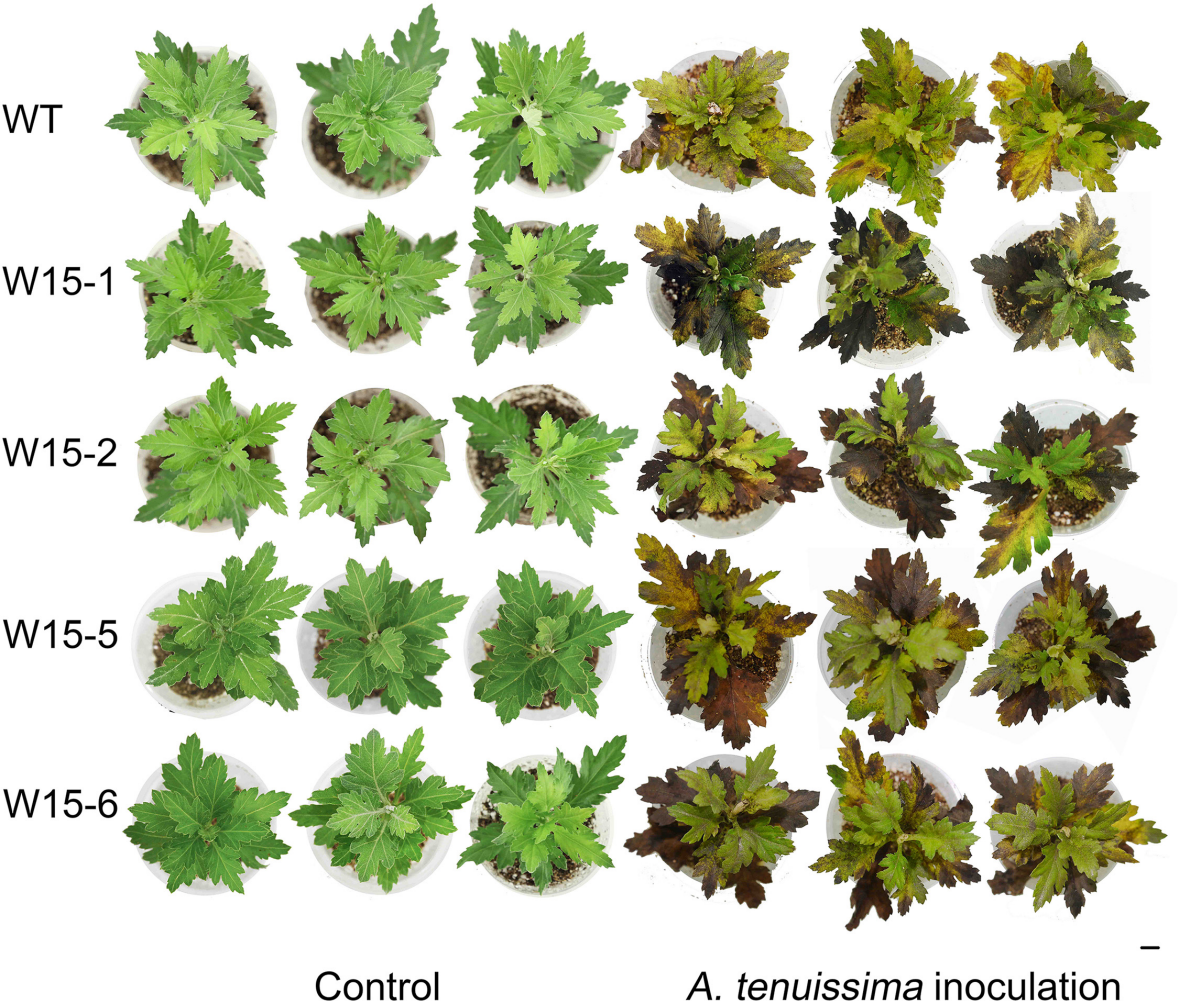
Phenotypic differences between transgenic chrysanthemum lines overexpressing *CmWRKY15* and wild-type ‘Jinba’ with *A*.*tenuissima* leaf spot infection. *A*. *tenuissima* was sprayed on seedlings derived from 20-day-old root cuttings taken from transgenic chrysanthemum and non-transgenic ‘Jinba’ (WT) chrysanthemum plants. Phenotypic effect of *A*. *tenuissima* exposure for 2 weeks compared to controls (given water for 2 weeks).

After exposure to *A*. *tenuissima* for 2 weeks, the surface area of the lesions on W15-1, W15-2, W15-5 and W15-6 plants was much larger compared to the control (Fig 4). Significant differences in *A*. *tenuissima* infection were clearly observable between the transgenic lines and wild type plants. The disease severity indexes (DSIs) of W15-1, W15-2 and WT were 57.01, 56.73 and 14.63, respectively (Table 1). The data revealed that the transgenic lines overexpressing *CmWRKY15* were susceptible (S) to black spot disease, while the non-transgenic plants were resistant (R). Necrosis was much more evident in the transgenic plants compared to the WT plants. Our results suggest that *CmWRKY15* overexpression enhanced the susceptibility of chrysanthemum to *A*. *tenuissima* attack.

**Table 1:** Responses of transgenic chrysanthemum lines overexpressing *CmWRKY15* and the wild-type line ‘Jinba’ to inoculation with *A*. *tenuissima*.

Materials	Disease severity index (DSI)*^a^	Host reaction*^b^
Wildtype ‘WT’	14.63 ± 0.63 a	R
*CmWRKY15-*1 ‘W15-1’	57.01 ± 1.33 b	S
*CmWRKY15-*2 ‘W15-2’	56.73 ± 1.65 b	S

Note: The means and standard deviations from ten independent experiments are shown.

*^a^Values followed by the same letter do not differ significantly according to Tukey’s multiple range test (P = 0.05).

*^b^Materials with a DSI of 0 are classified as immune (I), 1–10 are highly resistant (HR), 11–20 are resistant (R), 21–30 are moderately resistant (MR), 31–45 are moderately susceptible (MS), 46–70 are susceptible (S), and over 70 are highly susceptible (HS) to the disease.

### Analysis of ABA concentrations in transgenic chrysanthemum and wildtype ‘Jinba’

**Fig 5:**
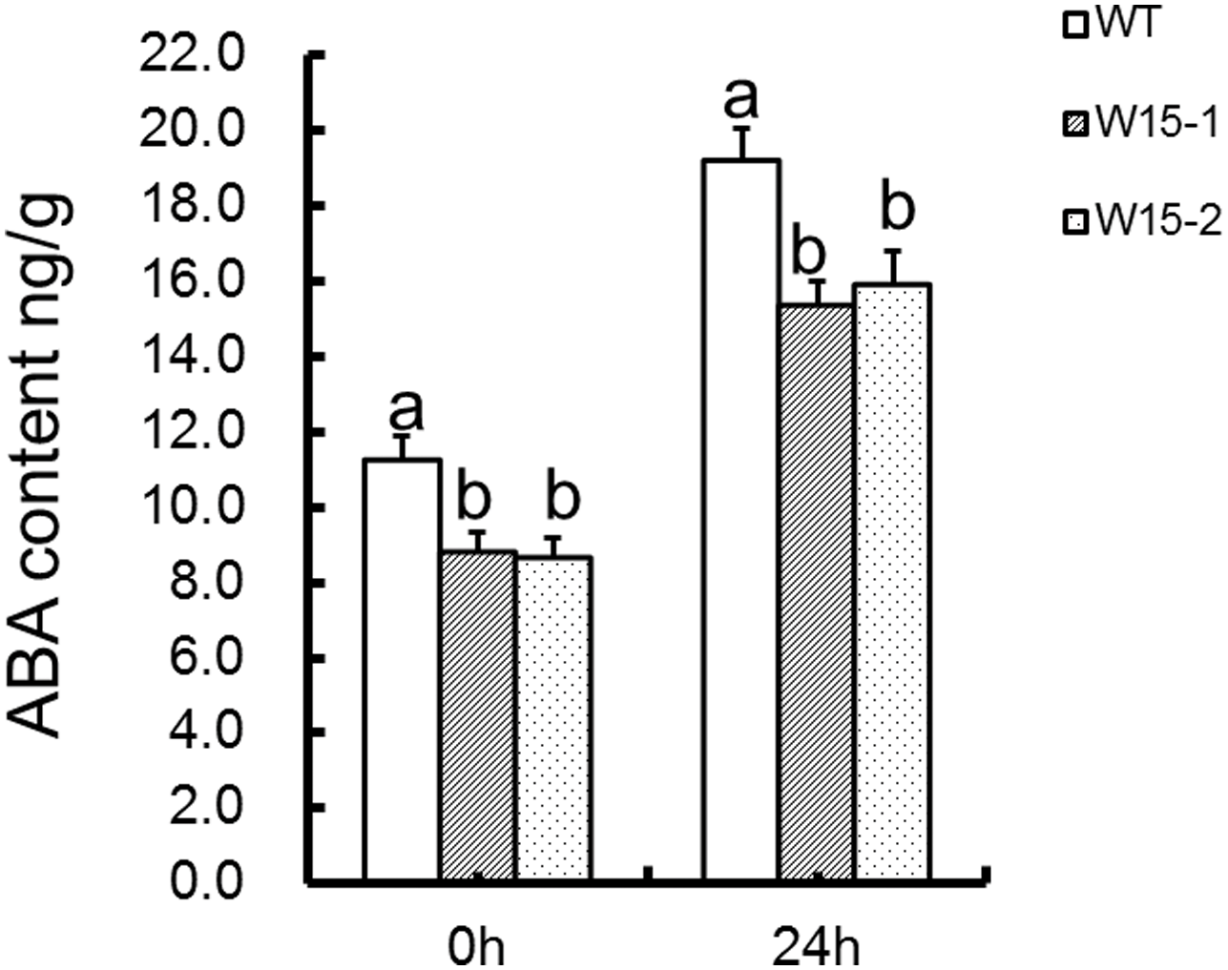
ABA concentrations in transgenic chrysanthemum and wild type ‘Jinba’ chrysanthemum. ABA concentrations of the control were assessed at 0 h, and ABA concentrations of plants exposed to *A*. *tenuissima* were assessed at 24 h.

Endogenous ABA content was measured in WT, W15-1 and W15-2 plants under normal conditions and 24 h after exposure to *A*. *tenuissima*. The results show that WT plants had a higher ABA content than transgenic lines overexpressing *CmWRKY15* (W15-1 and W15-2) (Fig 5) under normal conditions and after exposure to *A*. *tenuissima*. These data indicate that the ABA content increased in response to *A*. *tenuissima* and that *CmWRKY15* might inhibit endogenous ABA synthesis in transgenic plants.

### A putative mechanism by which *CmWRKY15* regulates ABA-responsive genes in response to *A*. *tenuissima* treatment

**Fig 6:**
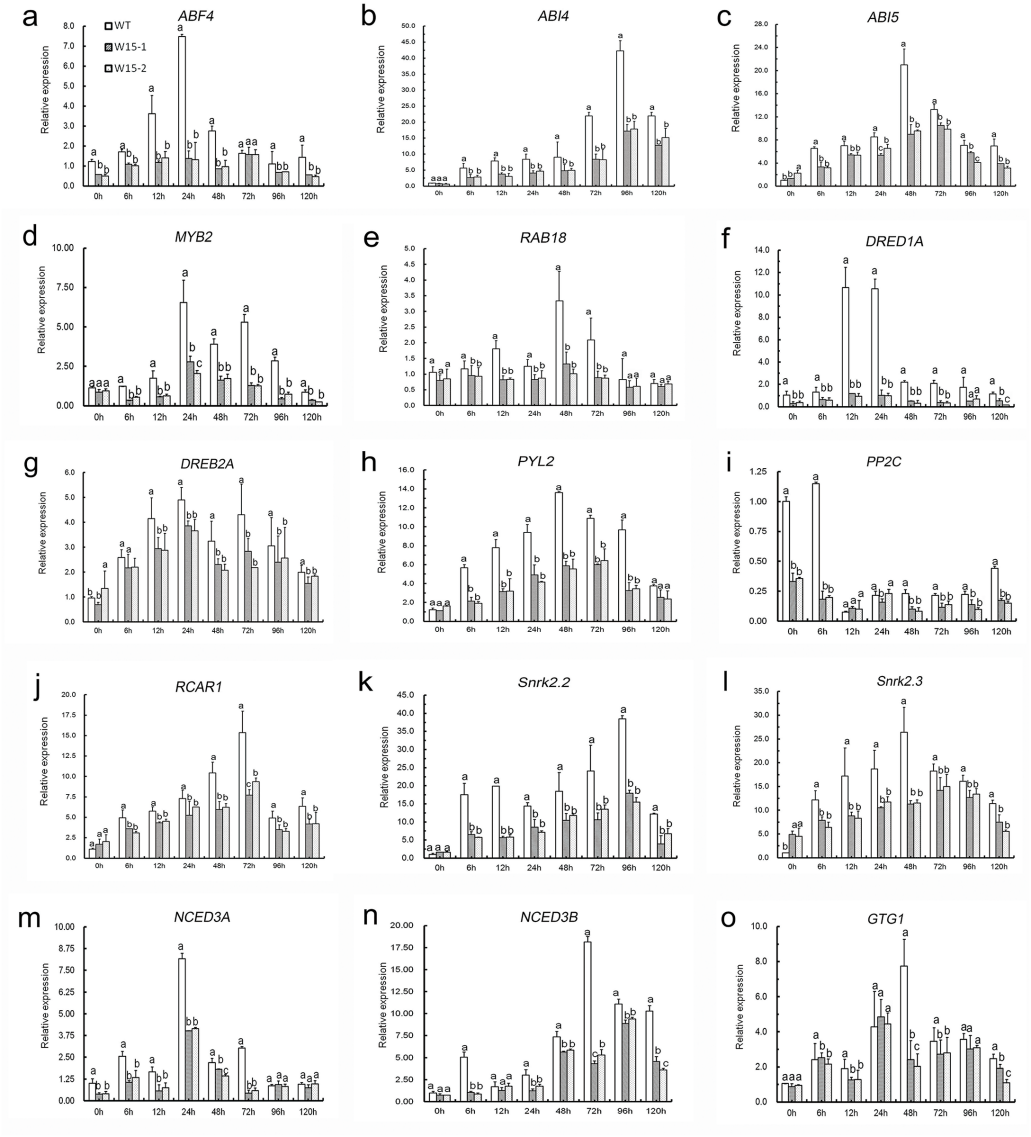
Expression of ABA-related genes in WT and the *CmWRKY15* transgenic lines (W15-1 and W15-2).

To identify the mechanisms by which *CmWRKY15* responds to *A*. *tenuissima*-induced stress, the expression levels of a set of ABA-related genes, including *ABF4*, *ABI4*, *ABI5*, *RAB18*, *DREB1A*, *DREB2A*, *PP2C*, *SnRK2*.*2*, *SnRK2*.*3*, *RCAR1*, *MYB2*, *PYL2*, *NCED3A*, *NCED3B*, *GTG1*, *AKT1*, *AKT2*, *KAT1*, *KAT2* and *KC1*, were compared between transgenic and WT plants (Fig 6, S1 Fig). Under non-stress conditions, the expression of most ABA-related genes was similar between transgenic lines and WT plants. Following *A*. *tenuissima* exposure, the transcription of the ABA-upregulated genes was impaired to varying degrees in *CmWRKY15*-overexpressing lines compared to the WT plants, while the ABA-downregulated genes were significantly upregulated in *CmWRKY15*-overexpressing lines. *ABF4*, *ABI4*, *ABI5*, *DREB1A*, *DREB2A*, *MYB2*, and *RAB18* were all downregulated significantly in the transgenic lines (Fig 6a–6g), and the expression level of *DREB1A* was induced nearly ten-fold in WT plants compared with that in the transgenic lines within 24 h of exposure to *A*. *tenuissima*. Moreover, the relative expression of *ABF4* was 7.49 in WT plants within 24 h of exposure, whereas this value was only 1.39 in W15-1 and 1.33 in W15-2. At their peaks, the expression levels of *ABI4*, *ABI5* and *MYB2* were upregulated 2–3-fold in the control compared with the transgenic lines. Before *A*. *tenuissima* inoculation, the expression of most ABA-related genes was not significantly different between transgenic chrysanthemum and WT plants. In contrast, after *A*. *tenuissima* infection, most genes were upregulated (Fig 6) or downregulated (S1 Fig) in WT plants compared with W15-1 and W15-2 plants. In addition to these genes, we evaluated the expression of two variants of NCED3 in chrysanthemum, *NCED3A* and *NCED3B*. The expression of these two genes was robustly induced by *A*. *tenuissima* infection, and their expression was significantly reduced in transgenic plants but not in WT plants, consistent with the observation of an increased ABA level in WT plants compared with transgenic lines overexpressing *CmWRKY15* (W15-1 and W15-2). Collectively, these results suggest that *CmWRKY15* might function as a transcriptional repressor that can facilitate *A*. *tenuissima* infection by directly or indirectly inhibiting the expression of ABA-responsive genes and genes related to ABA signaling, which can increase the expression of genes that are downregulated by ABA.

## Discussion

### Analysis of CmWRKY15 homologues

Three WRKY transcription factor homologues in *Arabidopsis*, WRKY18, WRKY40 and WRKY60, can interfere with pathogen defense. Several studies have demonstrated that CmWRKY15 homologues have a role in the pathogen response in a wide variety of species. For example, a gene involved in diverse stress-response pathways, *GhWRKY40* (isolated from cotton (*Gossypium hirsutum*)), negatively responded to invasion by the bacterial pathogen *Ralstonia solanacearum* by downregulating most defense-related genes [32]. However, *VpWRKY3* from *Vitis pseudoreticulata* was used to generate transgenic tobacco plants with enhanced resistance to *R*. *solanacearum* [33]. In addition, the expression of both *BnWRKY18* and *BnWRKY40* was significantly induced after treatment with the two necrotrophic fungal pathogens *Sclerotinia sclerotiorum* and *Alternaria brassicae* [34], which is consistent with earlier findings indicating that *A*. *tenuissima* induced *CmWRKY15* expression in chrysanthemum [13]. Moreover, a fungal pathogen-activated *elicitor* was found to immediately and transiently induce *PcWRKY4* expression in parsley cells (*Petroselinum crispum*) [35]. Recent research clearly shows that WRKY transcription factors homologous to CmWRKY15 participate in either biotic or abiotic stress-related responses. For example, *VpWRKY3* participates in the ABA signaling pathway and the salt stress response [33]. Similarly, *GarWRKY28* and *GarWRKY51* are salt-responsive genes from the salt-tolerant wild cotton species *Gossypium aridum* [36]. Moreover, *CrWRKY18* responds to JA in *Catharanthus roseus* [37]. Previous research clearly demonstrates that GhWRKY40, VpWRKY3 and PcWRKY4 are localized in the nucleus [32,33,35], in agreement with the finding that the CmWRKY15 protein is targeted to the nucleus; additionally, GarWRKY28 and GarWRKY51 proteins are predicted to reside in the nucleus [36]. CmWRKY15 was not found to exhibit transcriptional activation, unlike GhWRKY40 and VpWRKY3, which were found to function as transcriptional activators [32,33]. Together, these results suggest that the homologues of *CmWRKY15* in different species appear to have multiple roles in the response to pathogens. They also have diverse roles in controlling tolerance to biotic and abiotic stresses. Most homologous genes exhibit the same nuclear localization pattern, whereas their transcriptional activation patterns tend to differ. Our results demonstrating the response of *CmWRKY15* to *A*. *tenuissima* and its association with ABA-related genes represent fundamental data that will be useful in future research.

### Analysis of *CmWRKY15*-dependent regulation of ABA-responsive genes in response to *A*. *tenuissima* treatment

Quantitative real-time PCR analysis suggested that the expression of a set of ABA-related genes, including *ABF4*, *ABI4*, *ABI5*, *RAB18*, *DREB1A*, *DREB2A*, *PP2C*, *SnRK2*.*2*, *SnRK2*.*3*, *RCAR1*, *MYB2*, *PYL2*, *NCED3A*, *NCED3B*, *GTG1*, *AKT1*, *AKT2*, *KAT1*, *KAT2* and *KC1*, was altered to varying degrees in plants overexpressing *CmWRKY15* compared to control (WT) plants (Fig 6, S1 Fig). Among these genes, *ABF4*, *ABI4*, *ABI5*, *RAB18*, *DREB1A*, *DREB2A*, *PYL2*, *PP2C*, *SnRK2*.*2*, *SnRK2*.*3*, *RCAR1*, *MYB2*, *NCED3A*, *NCED3B* and *GTG1* exhibited responses that were synergistic with ABA-associated pathways or positively regulated ABA-associated pathways. Together with the observation that these genes were upregulated during exposure to *A*. *tenuissima*, we can conclude that ABA increases resistance to *A*. *tenuissima* by fine-tuning a set of ABA-related genes. Moreover, *AKT1*, *AKT2*, *KAT1*, *KAT2*, and *KC1* were downregulated by ABA, but they were upregulated in transgenic lines during exposure to *A*. *tenuissima*. Before *A*. *tenuissima* inoculation, the expression of most ABA-associated genes did not significantly differ between *CmWRKY15*-overexpressing plants and WT plants. In contrast, most genes were upregulated or downregulated in WT plants compared to W15-1 and W15-2 plants following exposure to *A*. *tenuissima*. A previous study showed that *CmWRKY15* was markedly upregulated in the presence of *A*. *tenuissima* inoculum [13]. Therefore, our results show that *CmWRKY15* in the transgenic overexpression lines has minimal effects under normal conditions. Conversely, *CmWRKY15* has a predominant role in regulating ABA-associated signaling in response to black spot disease.

### Analysis of ABF4, ABI4, ABI5, MYB2, RAB18, DREB1A and DREB2A transcript levels

In this study, the transcript levels of *ABF4*, *ABI4*, *ABI5*, *MYB2*, *RAB18*, *DREB1A* and *DREB2A* were significantly upregulated in WT plants compared to W15-1 and W15-2 plants following exposure to *A*. *tenuissima*. A chromatin co-immunoprecipitation (ChIP) assay using an antibody against WRKY40 demonstrated that AtWRKY40 directly targets several of these genes [18]. Note that ABI4, DREB1A, and DREB2A belong to a clade of Apetala-2 domain transcription factors, MYB2 is a MYB-related transcription factor, RAB18 is a rab-related protein, and ABI5 and ABF4 belong to a class of basic leucine zipper transcription factors. These four transcription factor families have been recognized as important players with positive roles in the ABA signaling network [18,38]. Our finding that these genes are upregulated in WT plants (Fig 6a–6g) suggests that *CmWRKY15* downregulates ABA-responsive genes and genes involved in ABA signaling in response to *A*. *tenuissima* infection. Moreover, *CmWRKY15* might indirectly regulate some ABA-responsive and ABA signaling genes via another pathway that represses *NCED3A* and *NCED3B*, leading to a decrease in ABA content.

### Analysis of *PYL2*, *PP2C*, *RCAR1*, *SnRK2*.*2*, and *SnRK2*.*3* transcript levels

We also noted that the transcript levels of *PYL2*, *PP2C*, *RCAR1*, *SnRK2*.*2* and *SnRK2*.*3* changed after exposure to *A*. *tenuissima* (Fig 6h and 6l). PYR/PYL/RCAR proteins, which are members of the START domain superfamily, have been identified as cytosolic ABA receptors [39]. When PYR/PYL/RCAR proteins bind to ABA, the inhibition of subclass III sucrose non-fermenting-1 (SNF1)-related protein kinase 2s (SnRK2s) can be alleviated by directly inhibiting type 2C protein phosphatases (PP2Cs) [40]. As a result, SnRK2s phosphorylate ABF4/ABI5 to synergistically modulate ABA-responsive genes involved in growth regulation and stress responses [39]. Given that *CmWRKY15* suppressed the expression of *ABF4/ABI5*, which function downstream of SnRK2s, we would expect the expression of *PYL2*, *PP2C*, *RCAR1*, *SnRK2*.*2* and *SnRK2*.*3* to be upregulated or unaltered in the *CmWRKY15*-overexpressing plants compared to the controls. Based on the evidence above, we can conclude that the crosstalk between *CmWRKY15* and ABA-mediated signaling pathways in plants during *A*. *tenuissima-*induced stress is complicated. Further research on the rudimentary mechanism underlying this crosstalk is necessary.

### Analysis of *GTG1* transcript levels

There was little variation in *GTG1* expression levels between *CmWRKY15*-overexpressing plants and WT plants after challenge with *A*. *tenuissima*; however, at 48 h, the expression of *GTG1* in the control plants was nearly 3-fold higher than that in the *CmWRKY15*-overexpressing plants (Fig 6o). GPCR-type G proteins are candidate plasma membrane-type ABA receptors, which are found on the cell surface and are required for ABA-induced promotion of stomatal closure [39]. Based on our data, we hypothesize that GTG1 is likely to interact with ABA to induce stomatal closure, thus protecting plants from *A*. *tenuissima* infection. At 48 h, *CmWRKY15* may have a negative effect on *GTG1* cooperation with ABA to limit *A*. *tenuissima* infection.

### Analysis of *NCED3A* and *NCED3B* transcript levels

NCED (9-cis-epoxycarotenoid dioxygenase) is an essential enzyme for the biosynthesis of ABA, which may be produced using two substrates: 9-cis-violaxanthin and/or 9’-cis-neoxanthin [41]. In *Arabidopsis*, NCEDs are predominantly localized in vascular tissues, which suggests that the ABA-activated stress pathway begins with a signal from the vascular tissues that induces stomatal closure [40]. *NCED* genes have been investigated to determine their roles in drought and salt stress. Additionally, a previous study revealed that *AtNCED3* is involved in infections involving pathogenic organisms, such as *Pseudomonas syringae pv*. *Tomato DC3000* and *B*. *cinerea* [42]. Here, using chrysanthemum, we evaluated the expression of two *NCED3* variants, *NCED3A* and *NCED3B*. We found that the concentration of endogenous ABA was higher in WT plants compared to transgenic lines overexpressing *CmWRKY15* (W15-1 and W15-2) (Fig 5). In addition, the expression of *NCED3A* and *NCED3B* was robustly induced by *A*. *tenuissima* infection and was significantly inhibited in transgenic plants but not in WT plants (Fig 6m and 6n). These results suggest that *CmWRKY15* may regulate ABA biosynthesis to a certain extent and interfere with ABA-induced stomatal closure to facilitate *A*. *tenuissima* infection. However, the complexity of the relationship between *CmWRKY15* and *NCED3* remains to be determined.

### Analysis of *AKT1*, *AKT2*, *KAT1*, *KAT2*, and *KC1* transcript levels

AKT1, AKT2, KAT1, KAT2, and KC1 form inward-rectifying K^+^ channels in *Arabidopsis* guard cells, and ABA can induce stomatal closure by inhibiting inward K^+^ channels [43]. Thus, it is obvious that the expression of *AKT1*, *AKT2*, *KAT1*, *KAT2*, and *KC1* was upregulated owing to the inhibition of ABA in transgenic lines during exposure to *A*. *tenuissima* (S1a–S1e Fig).

### Overexpression of *CmWRKY15* in chrysanthemum facilitated *A*. *tenuissima* infection

**Fig 7:**
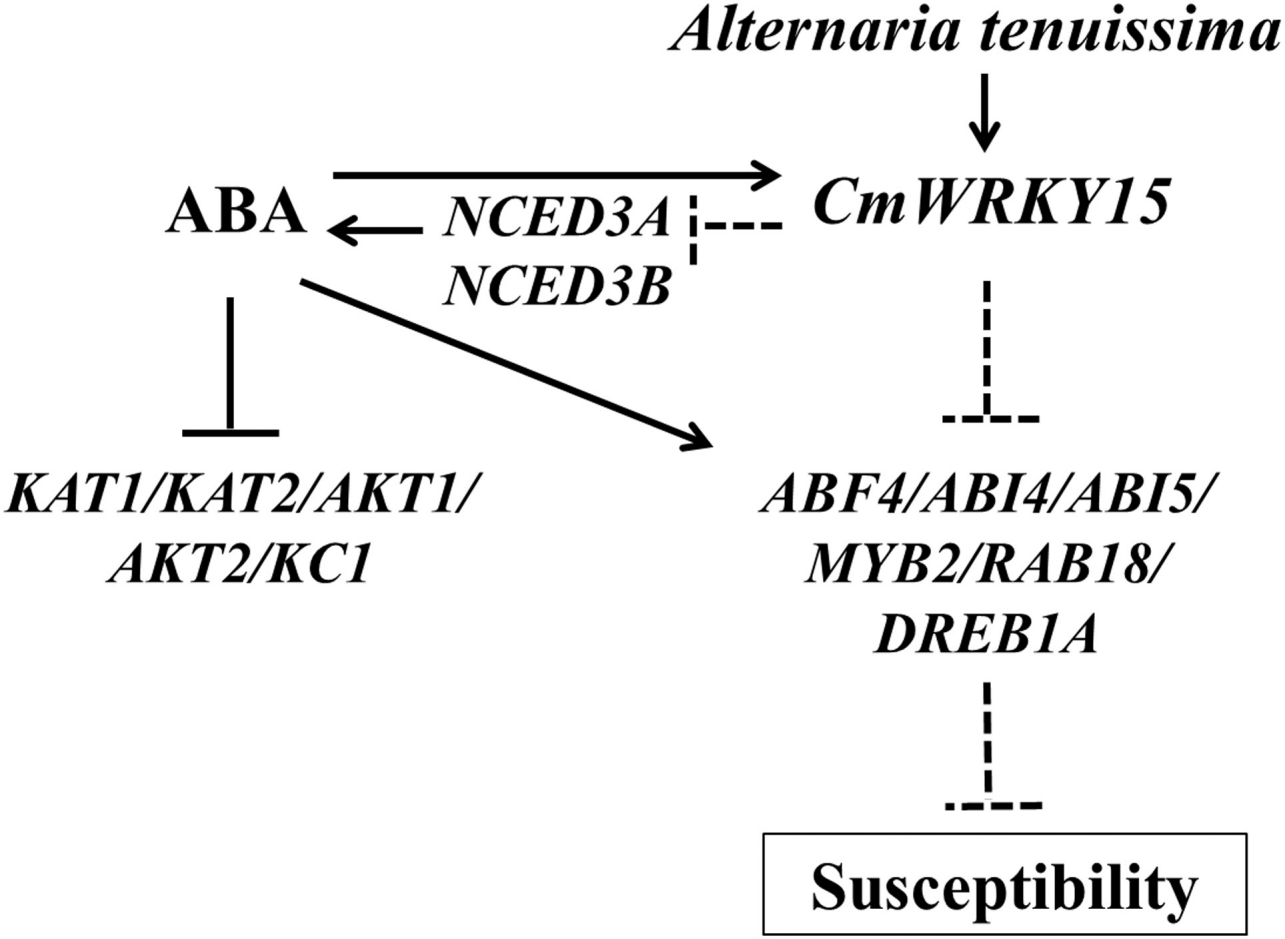
A putative model describing the effects of the interaction between *CmWRKY15* and ABA-associated genes on the defense response to *A*. *tenuissima* infection.

Based on the DSI data (Table 1) and the *A*. *tenuissima* treatment results (Fig 4), we can conclude that the transgenic *CmWRKY15* overexpression lines were susceptible to *A*. *tenuissima*. Taken together with our quantitative real-time PCR data (Fig 6), our results reveal that *CmWRKY15* can facilitate *A*. *tenuissima* infection via direct or indirect antagonistic regulation of ABA-responsive genes and ABA signaling genes, which upregulates the expression of genes that are downregulated by ABA (S1 Fig). In addition, *CmWRKY15* might indirectly regulate some ABA-responsive genes and ABA signaling genes via another pathway that represses *NCED3A* and *NCED3B* expression, leading to decreased ABA content (Fig 5). Here, we propose a model to describe the interactions that occur between *CmWRKY15* and ABA-responsive genes, ABA signaling genes and genes that are downregulated by ABA to modulate the *A*. *tenuissima* defense response (Fig 7). A previous study showed that ABA induced the expression of *CmWRKY15* [13], which supports a role for ABA in the regulation of CmWRKY15 expression. However, the basal mechanisms that fine-tune the interactions between *CmWRKY15* and ABA-responsive genes that modulate the *A*. *tenuissima* defense response remain unclear. Additional research on the integrative mechanisms of the signaling network will increase our understanding of the complicated crosstalk between *CmWRKY15* and ABA-mediated responses to *A*. *tenuissima* infection in chrysanthemum.

## Conclusions

CmWRKY15 was not transcriptionally activated in yeast cells. The results of subcellular localization experiments indicated that CmWRKY15 localized to the nucleus *in vivo*. A comparison of the disease severity index (DSI) data of *CmWRKY15*-overexpressing plants with the DSI data of controls indicated that *CmWRKY15* overexpression enhanced the susceptibility of chrysanthemum to *A*. *tenuissima* infection. Furthermore, an analysis of *CmWRKY15*-dependent regulation of ABA-responsive genes associated with *A*. *tenuissima* infection suggested that *CmWRKY15* might facilitate *A*. *tenuissima* infection by antagonistically regulating the expression of ABA-responsive genes and ABA signaling genes, either directly or indirectly, which could lead to the upregulation of genes that are downregulated by ABA.

## Supporting Information

S1 Fig Expression of ABA-downregulated genes in WT and the CmWRKY15 transgenic lines (W15-1 and W15-2). (TIF)

S2 Fig The W box motifs in the promoter regions of *ABI4*, *ABF4*, *NCED3*, *ABI5*, *RAB18*, *DREB2A*, *DREB1A* and *MYB2* genes. (TIF)

S1 Table Names and sequences of the primers used in this study. (PDF)
