# Supplementary figures and images for: CmWRKY15 Facilitates Alternaria tenuissima Infection of Chrysanthemum
Source: PLoS One. 2015 Nov 24;10(11):e0143349. doi: 10.1371/journal.pone.0143349 (PMC4658048; doi:10.1371/journal.pone.0143349)

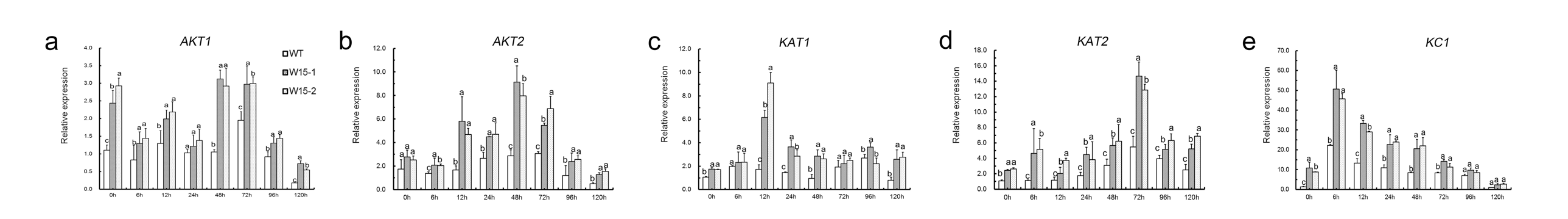

Supplement: S1 Fig — (TIF) [file pone.0143349.s001.tif]

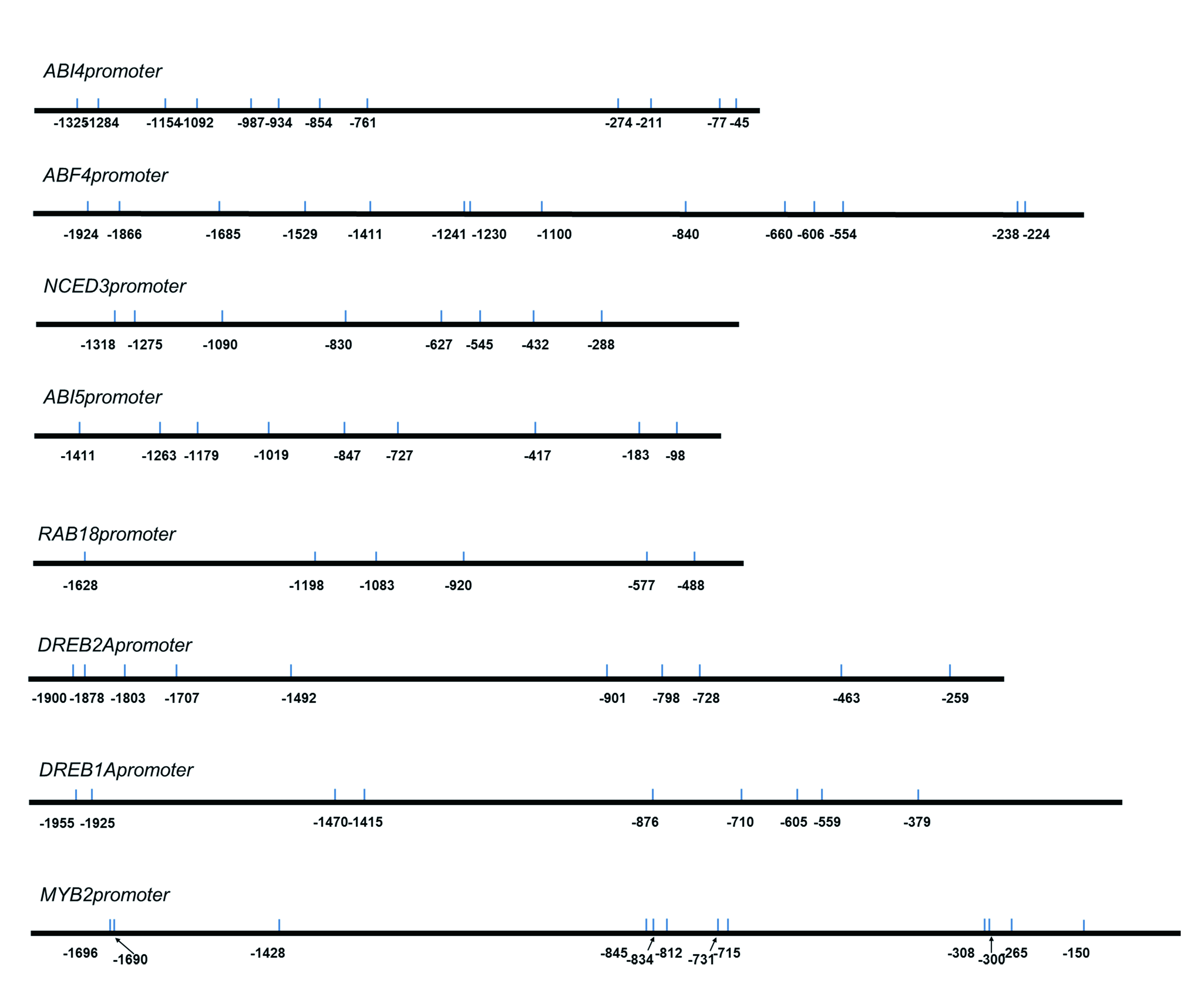

Supplement: S2 Fig — (TIF) [file pone.0143349.s002.tif]
