# Supplementary material for: CmWRKY15 Facilitates Alternaria tenuissima Infection of Chrysanthemum
Source: PLoS One. 2015 Nov 24;10(11):e0143349. doi: 10.1371/journal.pone.0143349 (PMC4658048; doi:10.1371/journal.pone.0143349)
Supplement: S1 Table — (PDF) [file pone.0143349.s003.pdf]

S1 Table Names and sequences of the primers used in this study.

Primer name	Sequence (5′ to 3′)
Oligo (dT)	AAGCAGTGGTATCAACGCAGAGTACTTTTTTTTTTTTTTT
dT-R	AAGCAGTGGTATCAACGCAGAGTAC
CmEF1α-F	TTTTGGTATCTGGTCCTGGAG
CmEF1α-R	CCATTCAAGCGACAGACTCA
CmABI4-F	GATCGTGGCTGAGAGACTCG
CmABI4-R	TACCCCACGTTCTTTGCCTC
CmABI5-F	CAGCAGCAGCAACAACTGTT
CmABI5-R	CTACAGGACTACCCAACGGC
CmDREB1A-F	CGGTTTTGGCTATGAGGGGT
CmDREB1A-R	TTCTTCTGCCAGCGTCACAT
CmDREB2A-F	GATCGTGGCTGAGAGACTCG
CmDREB2A-R	TACCCCACGTTCTTTGCCTC
CmMYB2-F	GGCCATGGACACTTGAGGAA
CmMYB2-R	ATTCCAACGGCCTTCACCAT
CmRAB18-F	TGTAGTGCAAAAACCCGGGT
CmRAB18-R	GTTGACCCCTCAGCAGTCAA
CmGTG1-F	CTGCAATAGCGGTGTCAGGA
CmGTG1-R	CTGACGAGCTGTGGAATGGT
CmPYL2-F	TCAATCCCACAAGGCCTCAC
CmPYL2-R	ACGAGGGATGTGCATGTGTT
CmRCAR1-F	GCCTTCCTGCCACTACAAGT
CmRCAR1-R	TTCCCTTCAGGCACATCGAC
CmSnRK2.2-F	ATTGTTGGATGGGAGCCCTG
CmSnRK2.2-R	GCTGGTGTACCAACTGTCGA
CmSnRK2.3-F	TAGCCAAGCTCCTAGGCTGA
CmSnRK2.3-R	GGGTAAGCTCCCACAAGCAT
CmPP2C-F	TAGTGATGGACTTTGGGATGTGG
CmPP2C-R	GCCAATGCCAACTTTGTCAGTAA
CmNCED3A-RT-F	AGTATGGTGGTGAGCCGTTGTATCTAC
CmNCED3A-RT-R	GCATTCACAATCTGGAGTTCGGACTTC
CmNCED3B-RT-F	CATACTTGGCGATTGCGGAACCAT
CmNCED3B-RT-R	GGCTCACCACCATACCTCTCATCAC
CmWRKY15-GATE-SAL-F	CGCGTCGACATGGTGGCTGCATCA
CmWRKY15-GATE-NOT-R	TTTGCGGCCGCGAACATACTTTGA
CmWRKY15-DL-F	TGCTCTTTCGCTCCAACCTG
CmWRKY15-DL-R	TTGTTCAACCAAAACCTCGTCA
CmAKT1-F	CATCGCTATCGGGCATCCTT
CmAKT1-R	CGACTATCCTTCCCACGACG
CmAKT2-F	TTGGTGCACTATGCTGCAGA
CmAKT2-R	GGGCTGTTTGGTCTGCATTG
CmKAT1-F	TCAATGGAGGTGCAATGGCT
CmKAT1-R	AGCGTCGCCTCTCTCAAAAA

CmKAT2-F	TGGCGGATCTTGCAAAGCTA
CmKAT2-R	CCAACAGCCTCGAAACTCCT
CmKC1-F	GGTTGTCCATGGCACCAGTA
CmKC1-R	TGACCGGATCGCTTTTGGAA
